# Altered *PTPN22* and *IL10* mRNA Expression Is Associated with Disease Activity and Renal Involvement in Systemic Lupus Erythematosus

**DOI:** 10.3390/diagnostics12112859

**Published:** 2022-11-18

**Authors:** Ilce Valeria Román-Fernández, Jesús René Machado-Contreras, José Francisco Muñoz-Valle, Alvaro Cruz, Diana Celeste Salazar-Camarena, Claudia Azucena Palafox-Sánchez

**Affiliations:** 1 Instituto de Investigación en Ciencias Biomédicas, Centro Universitario de Ciencias de la Salud, Universidad de Guadalajara, Guadalajara 44340, Mexico; 2 Laboratorio de Inmunología, Facultad de Medicina, Universidad Autónoma de Baja California, Mexicali 21000, Mexico; 3 Departamento de Reumatología, Hospital General de Occidente, Secretaría de Salud, Guadalajara 45170, Mexico

**Keywords:** systemic lupus erythematosus, *PTPN22*, *IL10*, lupus nephritis, SLEDAI

## Abstract

Systemic lupus erythematosus (SLE) is a complex autoimmune disease with very heterogeneous clinical behavior between affected individuals. Therefore, the search for biomarkers clinically useful for the diagnosis, prognosis, and monitoring of the disease is necessary. Here, we determined the association between *PTPN22*, *IL10*, *OAS2*, and *CD70* mRNA expression with the clinical characteristics and with the serum levels of IL-10, IFN-γ, and IL-17 in SLE patients. Forty patients with SLE and 34 control subjects (CS) were included, mRNA expression was determined by real-time qPCR and cytokine levels were quantified by a multiplex bead-based immunoassay. Compared to CS, SLE patients showed increased *IL10* mRNA and high IL-10 and IL-17 serum levels; in contrast, *PTPN22* mRNA and IFN-γ were decreased. *PTPN22* and *IL10* gene expression was negatively correlated with Mex-SLEDAI score and were notably downregulated in SLE patients with lupus nephritis. Interestingly, SLE patients with renal damage were the ones with the lowest levels of *PTPN22* and *IL10* mRNA and the highest SLEDAI scores. No associations were observed for *OAS2* and *CD70* mRNA and IL-10, IL-17, and IFN-γ. In conclusion, we suggest that the assessment of *IL10* and *PTPN22* mRNA could be useful for monitoring disease activity in SLE patients showing renal involvement.

## 1. Introduction

Systemic lupus erythematosus (SLE) is an inflammatory, chronic autoimmune disease characterized by the production of pathogenic autoantibodies directed against cytoplasmic and nuclear components [1]. The unrestricted activation of T and B cells may lead to the overproduction of autoantibodies, immune complex deposition, inflammatory cytokine release, and, eventually, organ damage [2]. The SLE patients show periods of remission alternate with mild to severe disease flares, leading to progressive organ damage [3]. Although not completely understood, it is known than SLE is a complex multifactorial disease that involves genetic susceptibility, environmental triggers, and hormonal factors for its development [1,3].

Among genetic factors, the protein tyrosine phosphatase non-receptor type 22 (*PTPN22*) is one of the most important genes associated with the development of SLE and other autoimmune diseases [4]. *PTPN22* encodes the lymphoid tyrosine phosphatase Lyp, which plays a key role in the regulation of the activation and effector functions of T [5] and B lymphocytes [6,7] as well as neutrophils [8], dendritic cells, and monocytes [9]. Accordingly, *PTPN22* expression has been studied in different autoimmune diseases, such as inflammatory bowel disease [10], rheumatoid arthritis [11], and SLE [12,13] with diverse results. In a previous study, we observed a decreased *PTPN22* mRNA expression in SLE patients, which was negatively correlated with disease activity. Interestingly, most SLE patients with severe disease activity had *PTPN22* mRNA expression levels which were nearly depleted [14]. These findings suggest that *PTPN22* expression could be included as a biological marker for monitoring disease activity in SLE patients.

Other immune-related genes have been found to be aberrantly expressed in SLE patients. In particular, a previous study proposed a T cell gene expression panel including the *OAS2*, *CD70*, and *IL10* genes as useful biomarkers for SLE discrimination from other autoimmune diseases and for the monitoring of disease activity [15]. The *OAS2* gene is an interferon-inducible gene that encodes an oligoadenylate synthetase which participates in the activation of RNase L for the degradation of double-stranded RNA in antiviral responses [16]. Altered enzymatic activity and the increased expression of the *OAS2* gene have been observed in SLE patients [17,18]. On the other hand, the *CD70* gene encodes a membrane protein that is part of the CD70/CD27 costimulatory pathway with a key role in T-B cell interactions for antibody production [19]. Similarly, the *IL10* gene, although encoding a cytokine with a regulatory function also has an important role in B cell activation and the stimulation of antibody production [20]. Both mRNA and protein products of the *CD70* and *IL10* genes have been found to be increased in SLE patients [19,21,22,23].

Additionally, previous studies have demonstrated the involvement of *PTPN22* and *CD70* genes in Th1 and Th17 cell polarization [24,25]. However, there is currently no information regarding the association of *PTPN22* and *CD70* mRNA gene expression with the levels of the cytokines primarily produced by these cell populations in SLE patients. 

Based on these data, the aim of the present study was to evaluate the association between *PTPN22*, *IL10*, *OAS2*, and *CD70* mRNA expression levels with the clinical characteristics and with the serum levels of IL-10, IFN-γ, and IL-17 in SLE patients.

## 2. Materials and Methods

### 2.1. Subjects

A total of forty patients with SLE classified according to the American College of Rheumatology (ACR) criteria were recruited from the Rheumatology Department of the Hospital General de Occidente, Guadalajara, Jalisco, Mexico. The Mexican version of Systemic Lupus Erythematosus Disease Activity Index (Mex-SLEDAI) [26] and the Systemic Lupus International Collaborating Clinics (SLICC) [27] damage index were applied to the SLE patients at the time of inclusion. According to the Mex-SLEDAI score, the disease activity was stratified as follows: inactive (0–2), mild-moderate (3–5), and severe (≥6) activity. The control subjects (CS) group included healthy individuals matched by age and gender. For cytokine analysis, a total of thirty-four CS were included, whereas qPCR assays were performed on eighteen of these subjects. All participants were from western Mexico.

A written informed consent was obtained from all study subjects. All procedures were performed following the ethical guidelines established in the 1964 Declaration of Helsinki and its later amendments or comparable ethical standards. The present study was also approved by the institutional Ethics and Research Committees (approval #449/16).

### 2.2. Laboratory Assessment

A complete blood count (CELL-DYN 3500R; Abbott Diagnostics, Lake Forest IL, USA) and the erythrocyte sedimentation rate (ESR, determined by the Wintrobe’s method) was performed on peripheral blood samples obtained from all SLE patients and CS included. For SLE patients’ samples, antinuclear autoantibodies (ANA) were determined by immunofluorescence standard procedures performed within the Hospital General de Occidente.

### 2.3. Real-Time Quantitative PCR Assay

Total RNA was isolated from peripheral blood leukocytes following the Chomczynski and Sacchi method using TRIzol™ reagent (Invitrogen Life technologies, Carlsbad, CA, USA). A reverse transcription to obtain complementary DNA (cDNA) was performed from a 1 μg of total RNA with Oligo(dT) primer and M-MLV reverse transcriptase protocol (Promega Corp., Madison, WI, USA); cDNA samples were stored at −80 °C until use. The *PTPN22*, *IL10*, *OAS2*, and *CD70* gene expression quantification was performed by the real-time quantitative PCR (qPCR) using the LightCycler^®^ Nano 2.0 (Roche Applied Science, Penzberg, Germany). Primers and hydrolysis probes were obtained from the Universal ProbeLibrary System Assay Desing platform, Roche (*PTPN22*: probe No. 78, cat. No. 04689011001; *IL10*: probe No. 67, cat. No. 04688660001; *OAS2*: probe No. 36, cat. No. 04687949001; *CD70*: probe No. 40, cat. No. 04687949001;). The glyceraldehyde 3-phosphate dehydrogenase (*GAPDH*) was used as a reference gene (cat. No. 05190541001). All samples were run as duplicates. The PCR efficiency for each gene was validated by running serial dilutions. The results obtained were analyzed by the 2^−ΔCq^ comparative method [28].

### 2.4. Quantification of IL-10, IL-17 and IFN-γ Serum Levels

Serum was obtained from a peripheral blood sample and stored at −80 °C until use. The serum levels of IL-10, IL-17, and IFN-γ cytokines were determined through a magnetic bead-based assay (Bio-Plex Pro™ Human Th17 Cytokine Panel; BIO-RAD, Hercules, CA, USA) and using the MAGPIX^®^-Luminex system (Luminex Corp, Austin, TX, USA). All samples were run in duplicate following manufacturer’s instructions. 

### 2.5. Statistical Analysis

Statistical analysis was carried out using SPSS version 12.0 (IBM Corporation, Armonk, NY, USA) and GraphPad Prism version 8.0 (GraphPad Software, San Diego, CA, USA) software. Comparisons between groups were performed using Kruskal–Wallis and Mann–Whitney U tests. Linear correlation coefficients were determined using Spearman’s correlation test. Statistical significance was considered at a *p* value < 0.05.

## 3. Results

### 3.1. Clinical Characteristics of SLE Patients

Thirty-nine women and one man diagnosed with SLE were included. According to laboratory results, SLE patients had significantly lower values of hemoglobin (12.5 ± 1.9 vs. 14 ± 1.3 g/dL, *p* < 0.05), hematocrit (38.1 ± 5.1 vs. 42 ± 3.4 %, *p* < 0.05), and total leucocytes (4.9 ± 3.3 vs. 6.4 ± 1.6 × 103/μL, *p* < 0.05), compared with the control group; on the other hand, ESR values were significantly higher in SLE patients (40 ± 10.5 vs. 20 ± 9.4 mm/h, *p* < 0.05). 

The main demographic and clinical features of SLE patients are summarized in Table 1. The mean age of patients was 31.8 ± 12 years, whereas the control group was 34 ± 12 years. For SLE patients, the median of disease duration was 4.0 (1.0–7.0) years and the median of disease activity was 4.31 (0.0–11.0) according to Mex-SLEDAI score, and the median damage score was 0.0 (0.0–1.0) according to SLICC damage index. Ninety-eight percent of patients were positive for ANA autoantibodies, with anti-dsDNA being the most frequent (63%). The most frequently presented clinical manifestations were hematologic (48%), followed by renal (30%) and mucocutaneous (30%), manifestations. All patients were under treatment with at least one immunosuppressive agent.

### 3.2. Levels of PTPN22, IL10, OAS2, and CD70 mRNA and IL-10, IL-17 and IFN-γ Cytokines in SLE Patients

**Figure 1:**
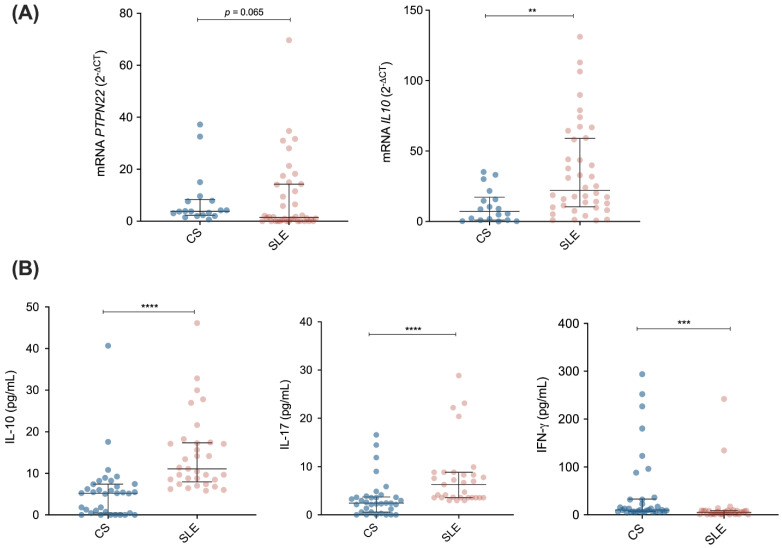
mRNA and cytokine levels in SLE patients and control subjects (CS). (**A**) mRNA expression of *PTPN22* and *IL10* genes determined by the 2^−ΔCq^ method in CS (*n* = 18) vs. SLE patients (*n* = 40); (**B**) Serum levels of IL-10, IL-17, and IFN-γ cytokines in CS (*n* = 34) vs. SLE patients (*n* = 31). Data are presented in median (p25–p75). Differences between groups were determined using Mann–Whitey U test. ** *p* < 0.01, *** *p* < 0.001, **** *p* < 0.0001.

Compared to CS, SLE patients showed a tendency toward decreased *PTPN22* mRNA levels (*p* = 0.065), whereas *IL10* mRNA gene expression was found to be significantly increased (*p* = 0.001, Figure 1A). There were no differences in *OAS2* and *CD70* mRNA gene expression between both groups (*p* > 0.05, Figure S1). Regarding cytokine levels, SLE patients presented significantly increased serum levels of IL-10 (11.08 vs. 5.24 pg/mL; *p* < 0.0001) and IL-17 (6.30 vs. 2.44 pg/mL; *p* < 0.0001) compared to CS. On the other hand, SLE patients showed significantly decreased levels of IFN-γ compared to CS (5.42 vs. 9.97 pg/mL, *p* < 0.001; Figure 1B).

### 3.3. Association between PTPN22, IL10, OAS2, and CD70 mRNA and IL-10, IL-17, and IFN-γ Cytokines with Clinical Characteristics in SLE Patients

**Figure 2:**
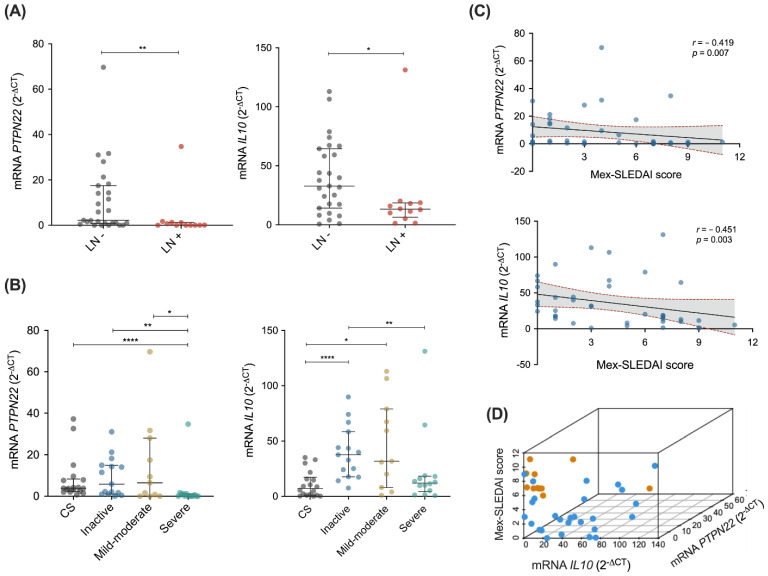
*PTPN22* and *IL10* gene expression in association with renal involvement and disease activity in SLE patients. (**A**) *PTPN22* and *IL10* mRNA in SLE patients without renal damage (LN-, *n* = 27) vs. patients with renal damage (LN+, *n* = 12), data are presented in median (p25–p75); (**B**) *PTPN22* and *IL10* mRNA according to Mex-SLEDAI disease activity groups: 0–2 inactive (*n* = 15), 3–5 mild-moderate (*n* = 11), and ≥6 severe (*n* = 14), data are presented in median (p25–p75); (**C**) correlation between *PTPN22* (top panel) and *IL10* (bottom panel) mRNA with Mex-SLEDAI score; (**D**) 3D plot showing Mex-SLEDAI score (y axis), *IL10* mRNA (x axis), and *PTPN22* mRNA (z axis) in SLE patients with renal damage (orange) and without renal damage (blue). Differences between groups were determined using the Mann–Whitney U test. Linear correlation coefficients were determined using Spearman’s correlation test. LN: lupus nephritis, CS: control subjects. * *p* < 0.05, ** *p* < 0.01, **** *p* < 0.0001.

The gene expression analysis of *PTPN22*, *OAS2*, *CD70*, and *IL10* according to the clinical features of SLE patients showed that patients with renal clinical manifestations had significantly decreased mRNA levels of *PTPN22* and *IL10* compared with patients without renal involvement (*p* = 0.007 and *p* = 0.033, respectively; Figure 2A). No significant differences were observed for *PTPN22* and *IL10* mRNA considering the presence of serositis, arthritis, hemolytic anemia, hematologic, or mucocutaneous manifestations (Table S1). Additionally, considering the role of the *PTPN22* gene product in T cell regulation, we evaluated the expression of *PTPN22* mRNA according to lymphopenia status, this was not significant (6.01 ± 9.69 vs. 10.0 ± 16.6; *p* = 0.76). No significant differences were observed for any of these clinical features, considering *OAS2* and *CD70* mRNA gene expression levels; this was also the case for IFN-γ, IL-10, and IL-17 serum levels (Tables S1 and S2).

The most significant associations observed in SLE patients were when considering disease activity. By stratifying SLE patients by disease activity groups according to the Mex-SLEDAI score, patients with severe disease activity showed the lowest *PTPN22* and *IL10* mRNA levels (*p* < 0.05), while those with the inactive or mild-moderate disease had similar or higher expression levels compared with CS (Figure 2B). Moreover, the mRNA gene expression levels of *PTPN22* and *IL10* showed a significant negative correlation with SLEDAI score (*r* = −0.419, *p* = 0.007 and *r* = −0.451, *p* = 0.003, respectively; Figure 2C). No differences were observed in *OAS2* and *CD70* mRNA gene expression as well as in IL-10, IL-17, and IFN-γ levels between Mex-SLEDAI activity groups (Figure S2). 

Considering the findings regarding the association of *PTPN22* and *IL10* gene expression with disease activity and with the presence of renal involvement in SLE patients by plotting these data into a 3D plot, we were able to confirm that most of the SLE patients who presented renal manifestations had the lowest levels of *PTPN22* and *IL10* mRNA as well as the highest Mex-SLEDAI scores (Figure 2D).

### 3.4. Correlation Patterns between PTPN22, IL10, OAS2, and CD70 mRNA and IL-10, IL-17, and IFN-γ Cytokine Levels in SLE Patients

**Figure 3:**
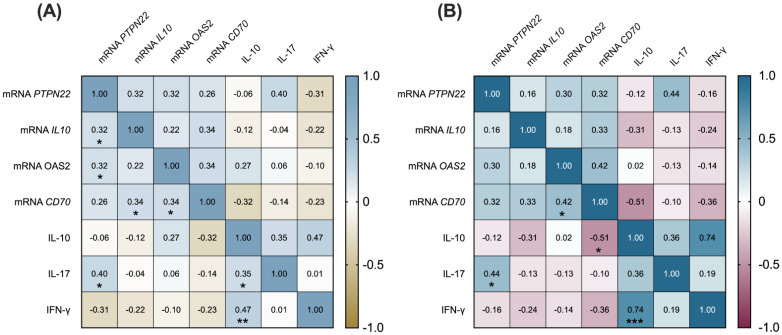
Correlations between *PTPN22*, *IL10*, *OAS2*, and *CD70* gene expression and IL-10, IL-17, and IFN-γ cytokine levels in SLE patients. (**A**) Correlation matrix showing correlation coefficient values considering all SLE patients; (**B**) Correlation matrix showing correlation coefficient values considering only active SLE patients (Mex-SLEDAI score > 2, *n* = 25). Linear correlation coefficients were determined using Spearman’s correlation test. * *p* < 0.05, ** *p* < 0.01, *** *p* < 0.001.

In order to determine the relation of *PTPN22*, *IL10*, *OAS2*, and *CD70* gene expression with IL-10, IL-17, and IFN-γ serum levels in SLE patients, a correlation analysis was performed. Positive correlations were observed between *PTPN22* mRNA with *IL10* mRNA (*p* = 0.044) and with *OAS2* mRNA (*p* = 0.043). Additionally, the *CD70* mRNA levels were positively correlated with those of *IL10* (*p* = 0.032) and *OAS2* (*p* = 0.032). Regarding cytokine levels, it was observed that IL-10 levels were positively correlated with those of IL-17 and those of IFN-γ (*p* = 0.046 and *p* = 0.005, respectively). Considering the association between mRNA gene expression and cytokine levels, a positive correlation was observed between *PTPN22* mRNA and IL-17 levels (*p* = 0.021; Figure 3A). 

Finally, we sought to determine whether these associations were to some extent related to the presence of clinical activity in SLE patients. Hence, a stratification of the patients according to active or inactive disease was carried out (Mex-SLEDAI score >2 and ≤2, respectively). We observed that the positive correlation between *CD70* and *OAS2* mRNA levels previously observed was stronger in patients with active disease (*p* = 0.034). This was also the case for the correlation observed between *PTPN22* mRNA and IL-17 serum levels (*p* = 0.046) as well as the one between IFN-γ and IL-10 (*p* = 0.0001). On the other hand, *CD70* mRNA was negatively correlated with IL-10 serum levels (*p* = 0.019; Figure 3B).

### 3.5. Levels of PTPN22, IL10, OAS2, and CD70 mRNA according to SLE Patient’s Treatment

**Figure 4:**
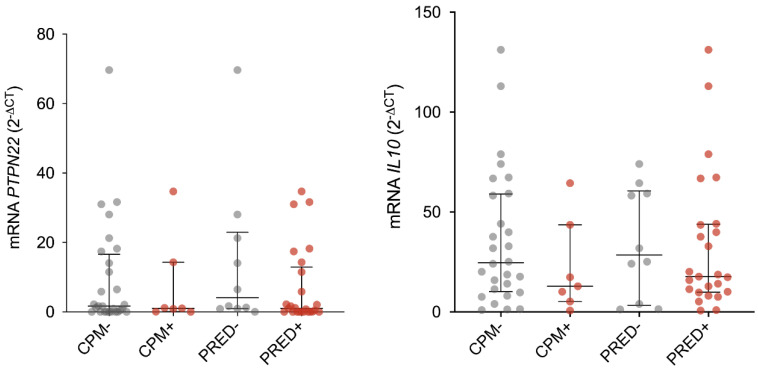
*PTPN22* and *IL10* mRNA expression according to SLE patient’s treatment. The figure shows the levels of *PTPN22* and *IL10* mRNA stratified by the use cyclophosphamide (CPM) or prednisone (PRED) in SLE patients. Data are presented in median (p25–p75). Statistical comparisons between groups were determined using the Mann–Whitney *U* test.

Because all the patients included in our study were under conventional therapy with immunosuppressants and glucocorticoids, we analyzed the expression of the genes according to the different treatments and no significant differences were found (Figure 4).

## 4. Discussion

SLE is a highly heterogeneous autoimmune disease, characterized by immune deregulation, resulting in altered T and B lymphocyte activation, autoantibody production, immune complex deposition, and organ damage. Patients with SLE have diverse clinical manifestations with remission and relapse periods [1,2,3]. Genetic factors are a key component in the etiology and pathogenesis of SLE, mainly involving genes with an immune-regulating function. In this context, *PTPN22* is one of the most important non-*HLA* genes [29], which has been associated with various autoimmune diseases, including rheumatoid arthritis [11], type 1 diabetes mellitus [30], and SLE [12,13]. In addition, the altered expression of several other immune-related genes has been observed in SLE patients [31]. It has been suggested that assessing the mRNA expression patterns of autoimmunity-associated genes could be clinically useful for the differentiation of SLE patients from other autoimmunity-affected individuals and for disease monitoring [15]. Taking this into account, in the present study, we evaluated the association between *PTPN22*, *OAS2*, *CD70,* and *IL10* mRNA expression levels with the clinical characteristics and serum levels of the functionally related cytokines IL-10, IL-17, and IFN-γ in SLE patients.

We found altered *PTPN22* an *IL10* gene expression in SLE patients. This agrees with some reports that have consistently identified a low *PTPN22* and an increased *IL10* expression in these patients. Hedrich et al. found an increased *IL10* mRNA expression in T cells isolated from SLE patients, which was associated with disease activity, and on a molecular level, with the hypomethylation of *IL10* promoter and increased STAT3 phosphorylation [32]. Moreover, Csiszár et al. reported higher numbers of *IL10* transcripts in unstimulated PBMCs from SLE patients compared to control subjects, identifying B cells and monocytes as the primary cellular sources [33]. In a study conducted by our research group, we found a significantly decreased *PTPN22* mRNA expression in SLE patients compared to healthy controls being negatively correlated with the Mex-SLEDAI score [14]. Conversely, no differences in *OAS2* and *CD70* mRNA were observed in SLE patients compared with CS. There are some studies that have reported a high *OAS2* and *CD70* gene expression in SLE patients [34,35]. Particularly, in a study it has been proposed a three gene-expression panel that included *IL10*, *OAS2,* and *CD70* genes that, according to the authors, were able to differentiate SLE patients from control subjects and from patients with other autoimmune diseases [15]. However, no consistent *OAS2* and *CD70* expression patterns have been found in SLE patients so far. These discrepancies may be due to differences in study design, including the quantification and interpretation methods of mRNA levels, the cell type analyzed, and the clinical characteristics of the patients included in each study. Consequently, this highlights the need for standardized and cost-effective technical procedures for a more homogeneous evaluation and comparison of gene expression profiles with potential clinical use in SLE patients.

We also analyzed the correlation patterns between mRNA gene expression and cytokine levels, we were able to observe that active SLE patients had fewer but stronger associations compared with those seen when all the patients were included in the correlation analysis. To the best of our knowledge, there is no information regarding combined mRNA and cytokine determinations for the identification of active SLE patients. Considering the results obtained in this study, it may be feasible to conduct studies with combined types of biomarkers for SLE patients’ classification and monitoring. In our study, despite the correlations observed in active SLE patients, we did not observe any association between *OAS2* and *CD70* mRNA expression with the presence of clinical manifestations; this was also the case for IL-10, IL-17, and IFN-γ levels. There are several studies that have determined cytokine profiles in SLE patients, mainly with the objective of SLE patients’ differentiation. Moreover, some of these have identified clusters of patients with high levels of certain cytokines and the presence of specific clinical manifestations [36,37,38,39]. In particular, there are studies reporting IL-10, IL17, and IFN-γ cytokine levels (by separate or as part of the same study) associated with active disease and have been proposed as useful cytokines for the classification of SLE patients with specific clinical characteristics, like high anti-dsDNA titers or the presence of renal involvement [36,40,41,42,43,44]. However, there are still no consistent results regarding cytokine profiling and SLE patients’ classification, which may be due to the well-known highly changing kinetics of cytokines or to technical differences associated with sample collection and storage [45].

The most significant observation derived from our study was the association between *PTPN22*- and *IL10*-altered mRNA expression with renal involvement and disease activity in SLE patients. Low mRNA levels of both gene products were associated with the presence of renal involvement and severe SLE clinical activity. Additionally, by plotting our data into a 3D plot we were able to confirm that most of the patients that presented renal involvement were the ones with the highest Mex-SLEDAI scores and with the lowest *PTNPN22* and *IL10* mRNA levels. The presence of renal damage was the only clinical manifestation that presented this kind of relation in the SLE group. Indeed, given that SLE is a clinically very heterogeneous disease, specific biomarkers for the diagnosis and monitoring of organ involvement have been proposed [46]. There are several factors that can influence the expression of genes related to the disease pathogenesis, which include drug effects, disease activity and the disease clinical heterogeneity. Additionally, it is known that glucocorticoids are able to increase the expression of IL-10 [47,48] and SLE patients usually present lymphopenia secondary to disease activity or related to the immunosuppressant treatment. Therefore, we analyzed the expression of the genes according to treatments and no differences were found. The above strengthens our findings that associate the expression of *PTPN22* and *IL10* related with disease activity. Although our observations need further validation, we suggest that *PTPN22* and *IL10* mRNA quantification in SLE patients may be useful for renal damage identification. We also suggest that prospective studies that determine the expression patterns of *PTPN22* and *IL10* over time are necessary since they would have the ability to provide valuable information on whether the expression kinetics of these genes is closely related to the development of renal damage in patients with SLE.

There are some limitations in our study, one of them is the relatively low number of samples used for mRNA and cytokine quantification; additionally, determinations were performed transversally. Therefore, it would be necessary to identify the kinetic profiles of *IL10* and *PTPN22* mRNA along the course of SLE and in a larger number of individuals in order to determine if the associations observed with renal activity are maintained and under which clinical scenarios this may change. It should also be noted that only the associations of the three cytokines were determined, of which only IL-17 was found to be associated with *PTPN22* mRNA in patients with active diseases. Therefore, it is not ruled out that the levels of other cytokines, especially those closely related to the biology of T and B lymphocytes, could have stronger associations with the studied genes.

Taking this together, we suggest that the assessment of *IL10* and *PTPN22* mRNA gene expression could be useful for monitoring SLE patients with severe disease activity who manifest some degree of renal damage.

## Figures and Tables

**Table 1:** Demographic and clinical characteristics of SLE patients (*n* = 40).

Male/female	1/39
Age (years) ^1^	31.8 ± 12
Disease duration (years) ^2^	4.0 (1.0–8.2)
**Clinical assessment**	
Mex-SLEDAI score ^2^	4.0 (1.0–7.0)
SLICC score	0.0 (0.0–1.0)
Autoantibody positivity ^3^	
ANA > 1:320	39 (98)
Anti-dsDNA	25 (63)
Anti-Sm	11 (28)
Anti-RNP	10 (25)
Anti-Ro	5 (13)
Anti-La	3 (8)
**Clinical manifestations ^3^**	
Hematologic ^a^	19 (48)
Renal ^c^	12 (30)
Mucocutaneous ^b^	12 (30)
Fatigue	11 (28)
Articular	7 (18)
Hemolytic anemia	7 (18)
Antiphospholipid syndrome	3 (8)
**Treatment ^3^**	
Prednisone	28 (70)
Chloroquine	20 (50)
Azathioprine	18 (45)
Cyclophosphamide IV ^d^	8 (20)
Hydroxychloroquine	8 (20)
Methotrexate	6 (15)

*Mex-SLEDAI*, Mexican version of the Systemic Lupus Erythematosus Disease Activity Index; *SLICC*, Systemic Lupus International Collaborating Clinics damage index. ^a^ Hematologic manifestations: presence of leucopenia, lymphopenia and/or thrombocytopenia; ^b^ Renal manifestations: creatinine (>5 mg/dL), proteinuria, hematuria, and cylinders; ^c^ Mucocutaneous manifestations: malar erythema, discoid lupus, oral ulcers, alopecia, and photosensitivity; ^d^ IV: intravenous.^1^ Data provided in mean ± SD; ^2^ Data provided in median (p25–p75); ^3^ Data provided in *n* (%).

## Data Availability

Not applicable.

## Supplementary Materials

The following supporting information can be downloaded at: https://www.mdpi.com/article/10.3390/diagnostics12112859/s1, Figure S1: Gene expression levels of *OAS2* and *CD70* in CS and SLE patients; Table S1: Association of *PTPN22*, *OAS2*, *IL10* and *CD70* mRNA expression levels with the clinical characteristics of SLE patients; Table S2: Association of IL-10, IL-17, and IFN-γ serum levels with the clinical characteristics of SLE patients; Figure S2: mRNA and cytokine levels in SLE patients according to clinical activity.
