# Supplementary material for: Altered PTPN22 and IL10 mRNA Expression Is Associated with Disease Activity and Renal Involvement in Systemic Lupus Erythematosus
Source: Diagnostics (Basel). 2022 Nov 18;12(11):2859. doi: 10.3390/diagnostics12112859 (PMC9689646; doi:10.3390/diagnostics12112859)
Supplement: Supplementary file 1 [file diagnostics-12-02859-s001.zip › diagnostics-1986375-supplementary.pdf]

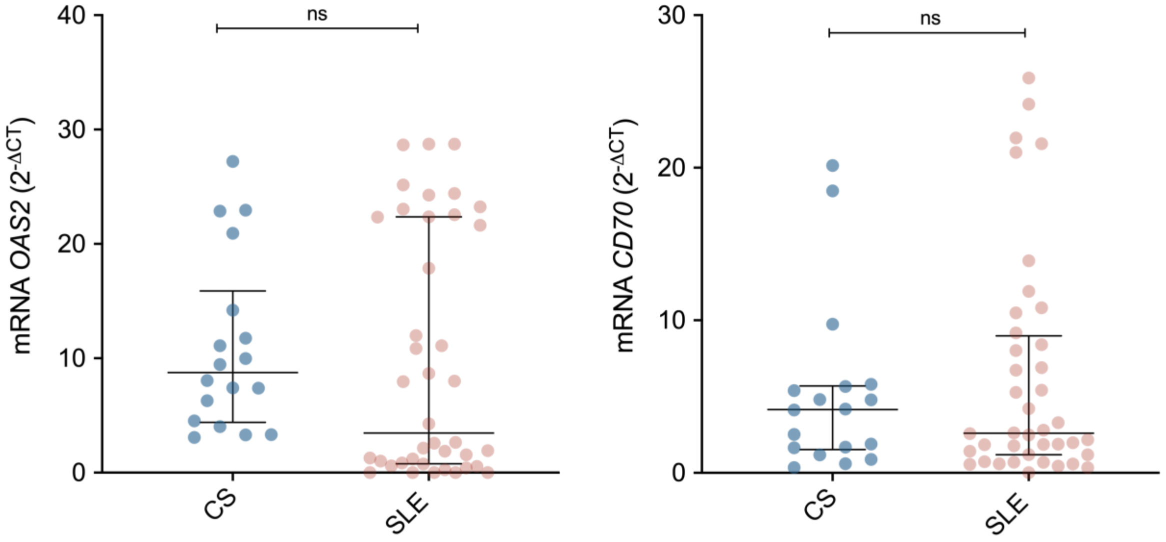

Supplementary Figure S1. Gene expression levels of *OAS2* (left panel) and *CD70* (right panel) in CS and SLE patients. Data are presented in median (p25-p75). Statistical comparisons between groups were determined using the Mann–Whitney *U* test.

Supplementary Table S1. Association of *PTPN22*, *OAS2*, *IL10,* and *CD70* mRNA expression levels with the clinical characteristics of SLE patients.

	PTPN22	***IL10***	***OAS2***	***CD70***
**Renal activity**				
Positive (n=12)	0.04 (0.001-1.23)	13.2 (6.41-18.5)	3.42 (1.37-19.0)	2.57 (0.69-11.0)
Negative (n=27)	2.20 (0.79-17.4)	32.9 (14.1-64-4)	8.01 (0.59-23.0)	2.49 (1.19-9.18)
p	**0.007**	**0.033**	0.84	0.99
**Serositis**				
Positive (n=3)	0.87 (0.001-17.4)	64.4 (11.4-78.9)	2.57 (1.92-22.3)	1.42 (0.69-25.9)
Negative (n=36)	1.43 (0.03-13.4)	19.4 (9.81-44.0)	6.11 (0.63-22.5)	2.60 (1.19-8.89)
p	0.82	0.35	0.90	0.94
**Hematologic**				
Positive (n=19)	1.16 (0.03-17.4)	18.1 (11.4-59.2)	2.65 (1.01-23.0)	1.97 (0.69-11.9)
Negative (n=20)	1.43 (0.03-11.0)	24.6 (7.65-53.6)	6.14 (0.63-20.7)	2.60 (1.79-8.56)
p	0.92	0.94	0.61	0.79
**Mucocutaneous**				
Positive (n=11)	1.69 (1.16-31.0)	18.7 (3.99-37.6)	11.1 (1.01-23.0)	4.21 (1.19-11.9)
Negative (n=28)	0.91 (0.02-11.0)	22.1 (11.9-59.0)	2.61 (0.63-22.4)	2.32 (0.85-8.61)
p	0.17	0.29	0.39	0.61
**Arthritis**				
Positive (n=7)	1.16 (0.02-11.5)	7.51 (3.99-78.9)	12.0 (0.59-23.2)	8.02 (1.86-21.6)
Negative (n=32)	1.43 (0.03-14.2)	22.1 (13.0-54.7)	2.61 (0.78-22.2)	2.32 (0.70-6.85)
p	0.93	0.51	0.40	0.11
**Hemolytic anemia**				
Positive (n=7)	1.69 (0.87-18.2)	17.7 (1.39-64.4)	1.92 (1.20-24.3)	1.42 (0.73-10.5)
Negative (n=31)	1.16 (0.01-14.1)	20.1 (11.4-44.1)	7.96 (0.55-22.4)	2.63 (1.19-8.02)
p	0.44	0.71	0.96	0.46

Data are presented in median (p25-p75). Statistical comparisons between groups were determined using the Mann–Whitney *U* test. The statistical significance was considered at $p < 0.05$.

Supplementary Table S2. Association of IL-10, IL-17, and IFN-γ serum levels with the clinical characteristics of SLE patients.

	IL-10 (pg/mL)	IL-17 (pg/mL)	IFN-γ (pg/mL)
**Renal activity**			
Positive (n=11)	8.55 (7.02-17.27)	4.15 (3.57-7.78)	5.42 (1.11-13.9)
Negative (n=22)	13.80 (8.82-17.65)	7.41 (3.57-12.54)	7.22 (1.24-9.06)
p	0.28	0.35	0.78
**Serositis**			
Positive (n=3)	9.65 (5.83-17.13)	3.57 (3.57-20.38)	5.42 (5.42-8.31)
Negative (n=30)	11.24 (8.22-17.65)	6.84 (3.57-8.85)	6.14 (1.15-9.28)
p	0.48	0.65	0.95
**Hematologic**			
Positive (n=19)	13.43 (8.82-17.27)	7.58 (3.57-8.85)	6.14 (1.15-13.90)
Negative (n=14)	9.24 (6.70-18.48)	4.44 (3.57-9.12)	6.87 (1.25-9.06)
p	0.38	0.49	0.88
**Mucocutaneous**			
Positive (n=8)	13.80 (8.78-27.56)	7.94 (4.35-54.48)	71.47 (2.76-226.1)
Negative (n=25)	10.45 (7.41-17.20)	4.72 (3.57-8.85)	5.42 (1.21-8.49)
p	0.36	0.25	0.08
**Arthritis**			
Positive (n=6)	9.24 (6.55-28.41)	6.75 (3.57-226.0)	8.71 (4.40-205.0)
Negative (n=27)	11.40 (8.49-17.27)	6.70 (3.57-8.85)	5.42 (1.15-8.67)
p	0.67	0.61	0.18
**Hemolytic anemia**			
Positive (n=5)	14.16 (8.34-17.29)	6.30 (3.86-21.75)	8.31 (3.29-8.68)
Negative (n=27)	11.08 (7.41-18.26)	6.97 (3.57-8.85)	5.42 (1.15-9.78)
p	0.89	0.83	0.81

Data are presented in median (p25-p75). Statistical comparisons between groups were determined using the Mann–Whitney *U* test. The statistical significance was considered at $p < 0.05$.

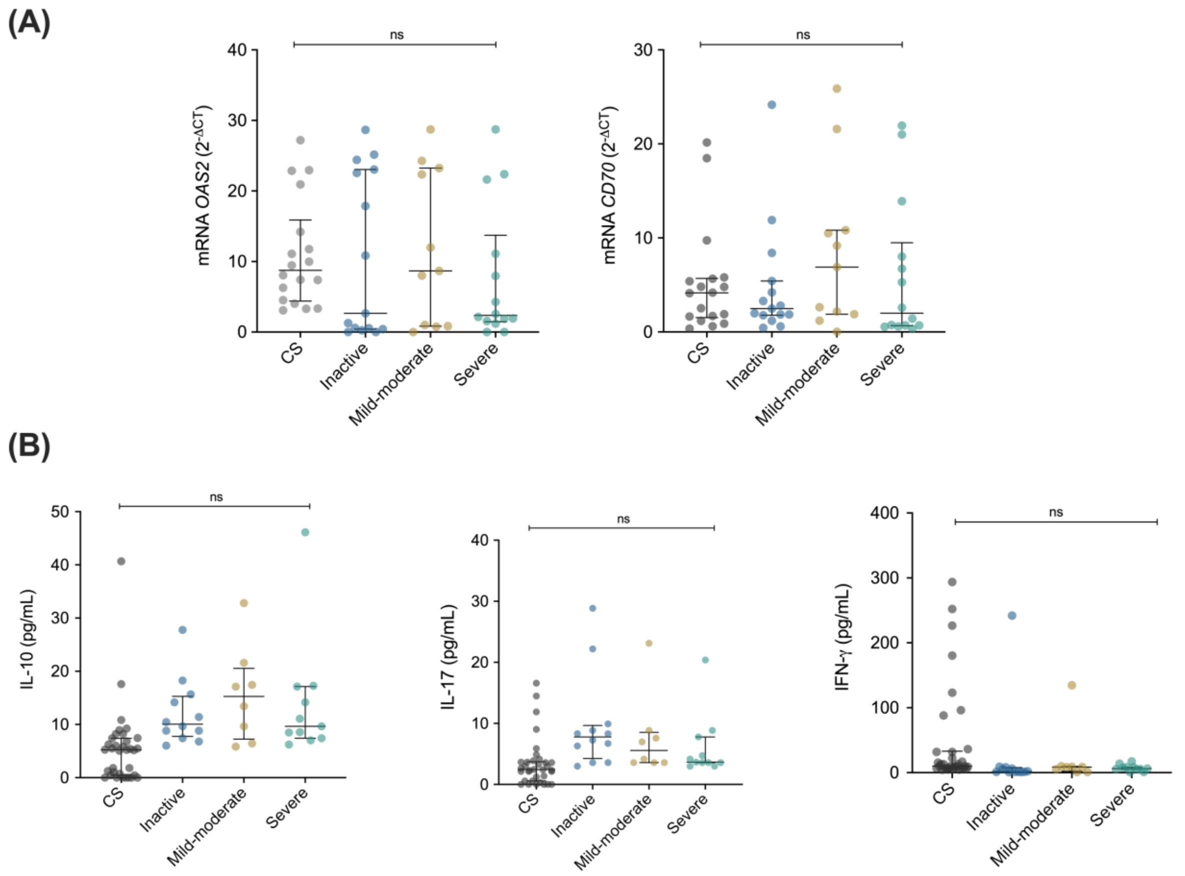

Supplementary Figure S2. mRNA and cytokine levels in SLE patients according to clinical activity. (**A**) Gene expression levels of *OAS2* and *CD70* as well as (**B**) IL-10, IL-17, and IFN-γ serum levels in SLE patients are shown according to SLEDAI score clinical activity groups. Data are presented in median (p25-p75). Statistical comparisons were determined using Kruskal–Wallis test.
